# *DC-SIGN* (*CD209*) Promoter −336 A/G Polymorphism Is Associated with Dengue Hemorrhagic Fever and Correlated to DC-SIGN Expression and Immune Augmentation

**DOI:** 10.1371/journal.pntd.0000934

**Published:** 2011-01-04

**Authors:** Lin Wang, Rong-Fu Chen, Jien-Wei Liu, Ing-Kit Lee, Chiu-Ping Lee, Ho-Chang Kuo, Shau-Ku Huang, Kuender D. Yang

**Affiliations:** 1 Department of Pediatrics, Chang Gung Memorial Hospital-Kaohsiung Medical Center (CGMH-KMC), Kaohsiung, Taiwan; 2 Department of Medical Research, CGMH-KMC, Kaohsiung, Taiwan; 3 Division of Infectious Diseases, Department of Internal Medicine, CGMH-KMC, Kaohsiung, Taiwan; 4 Johns Hopkins Asthma and Allergy Center, Baltimore, Maryland, United States of America; 5 Graduate Institute of Clinical Medical Sciences, Chang Gung University College of Medicine, Kaohsiung, Taiwan; Institute of Tropical Medicine (NEKKEN), Japan

## Abstract

**Background:**

The C-type lectin DC-SIGN (CD209) is known to be the major dengue receptor on human dendritic cells, and a single nucleotide polymorphism (SNP) in the promoter region of *CD209* (−336 A/G; rs4804803) is susceptible to many infectious diseases. We reason that variations in the DC-SIGN gene might have a broad influence on viral replication and host immune responses.

**Methods and Findings:**

We studied whether the rs4804803 SNP was associated with a susceptibility to dengue fever (DF) and/or dengue hemorrhagic fever (DHF) through genotyping analysis in a Taiwanese cohort. We generated monocyte-derived dendritic cells (MDDCs) from individuals with AA or AG genotype of rs4804803 to study the viral replication and immune responses for functional validation. A total of 574 DNA samples were genotyped, including 176 DF, 135 DHF, 143 other non-dengue febrile illnesses (OFI) and 120 population controls. A strong association between GG/AG genotypes of rs4804803 and risk of DHF was found when compared among DF, OFI and controls (*p* = 0.004, 3×10^−5^ and 0.001, respectively). The AA genotype was associated with protection against dengue infection compared with OFI and controls (*p* = 0.002 and 0.020, respectively). Moreover, MDDCs from individuals with AG genotype with a higher cell surface DC-SIGN expression had a significantly higher TNFα, IL-12p40, and IP-10 production than those with AA genotype in response to dengue infection. However, the viral replication in MDDCs with AG genotype was significantly lower than those with AA genotype. With both genotypes, MDDCs revealed an increase in viral replication following the addition of anti-IP-10 neutralizing antibody.

**Conclusions/Significance:**

The rs4804803 SNP in the *CD209* promoter contributed to susceptibility to dengue infection and complication of DHF. This SNP with AG genotype affects the cell surface DC-SIGN expression related to immune augmentation and less viral replication.

## Author Summary

Dengue fever (DF) is an arthropod-borne disease that is prevalent in tropical and subtropical regions of the world. DC-SIGN [dendritic cell-specific intercellular adhesion molecule 3 (ICAM-3)-grabbing non-integrin] is a major receptor for dengue infection. DC-SIGN, also called CD209, expresses on dendritic cells (DCs) that bind to ICAM-3, which is expressed on T cells to facilitate the initial interaction between DCs and T cells. Variations in the *CD209* promoter (−336 A/G; rs4804803) genotype are involved in the pathogenesis of human infectious diseases. Here we found that patients with dengue hemorrhagic fever (DHF) had a higher frequency of the AG or GG genotype of rs4804803 than DF or controls. Functional studies determined that monocyte-derived DCs (MDDCs) from individuals with AG genotype had significantly higher cell surface DC-SIGN expression, associated with higher TNFα, IL-12p40, and IP-10 production, but lower viral replication than those with AA genotype. An increase in DEN-2 replication in MDDCs was observed following the addition of anti-IP-10 neutralizing antibody. These findings highlight the fact that the rs4804803 SNP in the *CD209* promoter is associated with DHF and correlated to DC-SIGN expression and immune augmentation.

## Introduction

Dengue viruses (DEN) are arthropod-borne flaviviruses that cause dengue fever (DF) with significant morbidity and mortality in tropical and subtropical regions of the world. There are four serotypes of dengue viruses (DEN types 1–4). Classic DF is a self-limited illness characterized by fever, headache, myalgia, arthralgia, and abdominal pain. Since the 1950s, a more severe form of the disease, dengue hemorrhagic fever (DHF), has been recognized worldwide [1]. Patients who develop DHF typically have initial symptoms similar to those in DF patients, but develop cytokine-storm-like plasma leakage manifested by hemoconcentration, thrombocytopenia, ascites, and pleural effusion near the defeverence stage [2]. DHF pathogenesis has been attributed to viral virulence versus immune enhancement; however, that has been the subject of debate for many years [2], [3].

The innate immune system is the first line of host defense against pathogens and is involved in early recognition and uptake of microbes by the host's professional phagocytes such as dendritic cells (DCs) and macrophages, through germline-encoded receptors, known as pattern recognition receptors (PRRs) [4]. These PRRs recognize microbial antigens and initiate innate immune responses followed by adaptive immunity [5]. PRRs are involved in phagocytosis, antigen presentation, and they trigger intracellular signaling and cytokine secretion [5]. PRR polymorphisms may therefore affect virus entry, replication, and immunity.

Among the PRRs, the CD209 molecule, also known as DC-SIGN (dendritic cell-specific intercellular adhesion molecule-3 grabbing non-integrin), plays an important role in the early interaction of a pathogen with a dendritic cell [6]–[8] and has a key role in DC-T cell interaction [9], DC migration [10], [11], and pathogen uptake [12]. DC-SIGN is organized into three domains, the N-terminal domain is located in the cytoplasm, the transmembrane domain anchors to the cytoplasmic membrane, and the extracellular domain consists of a neck region formed by seven highly conserved 23 amino acid repeats and a carbohydrate domain for pathogen binding [13].

The *CD209* gene is located on chromosome 19p13.2–3 and is highly polymorphic. Numerous single nucleotide polymorphisms (SNPs) have been reported [14]–[18]. One of these SNPs represents a guanine (G) to adenine (A) transition at position −336 within the *CD209* gene promoter (rs4804803). This variant has been associated with an increased risk for parenteral acquisition of human immunodeficiency virus (HIV) infection [15], severity of dengue infection [16], and confered high susceptibility to tuberculosis in a South African cohort [17]. Nevertheless, Vannberg *et al.* found that G variant allele of rs4804803 was associated with protection against tuberculosis in individuals from sub-Saharan Africa [18]. This variant affects *CD209* promoter activity with multiple transcription factor binding sites for the Sp1/GATA1/CACCC- and CAC-binding transcription factors in a transfection study [16]. As an *in vitro* study of promoter activity might not reflect an actual functional association, we aimed to test whether the rs4804803 SNP in the promoter region of *CD209* was associated with the susceptibility to DF and/or DHF in Taiwanese, and whether monocyte-derived DCs from humans with various genotypes of rs4804803 would reveal differences in DC-SIGN membrane expression and implicate the viral replication and immune reactions after DEN infection.

## Materials and Methods

### Ethics statement and subjects studied

This study was approved by the Institution Review Board (IRB) of Chang Gung Memorial Hospital-Kaohsiung Medical Center, Taiwan. The dengue patients were recruited as described previously in the 2002–2003 DEN-2 outbreak in Taiwan [19]–[23]. A larger retrospective cohort was designed and re-approved by an additional IRB review (Document No.: 97-2111B). To validate cell surface expression and immune functions of rs4804803 SNP, we obtained informed consent to collect blood leukocytes from normal volunteers with AA or AG genotypes of rs4804803.

### Definition of cases and controls

DEN infection was confirmed by clinical dengue symptoms and signs along with detection of DEN-2 RNA by quantitative RT-PCR in blood, detection of IgM to DEN or at least a 4-fold increase in dengue-specific hemagglutination inhibition titers in convalescent serum compared with that in acute-phase serum [20], [21]. In those with DEN-2 infection, blood was drawn at least once a day subsequent to admission into the hospital to measure the platelet counts and hematocrit levels. A Chest X-ray and abdominal ultrasonography were performed routinely in individuals without evidence of hemoconcentration or hypoalbuminemia to refine the differential diagnosis of DHF vs. DF based on pleural effusion or ascites. A clinical diagnosis of DHF was assigned according to the DHF criteria of the World Health Organization (WHO); including a reduced platelet count (<100,000/mm^3^), petechiae, hemorrhagic manifestation, and plasma leakage showing hemoconcentration (peak hematocrit ≥20% above the mean for the population, or an increase in hematocrit of 20% or more), pleural effusion, ascites, or hypoalbuminemia [24]. Patients with DF were defined by detectable DEN-2 RNA by RT-PCR or DEN-specific IgM, but without evidence of DHF. Primary or secondary DEN infections were identified using previously established serologic criteria for IgM/IgG ELISAs [19].

Patients with other non-dengue febrile illnesses (OFI) were defined by febrile illness with no detectable DEN-specific IgM, no detectable DEN RNA, and no obvious or reported bacterial etiology for their illness during the same study period. Population controls were healthy, unrelated volunteers from the same community, with neither signs nor previous history of dengue infection, with a DEN IgG sero-positive rate of 1.37% (1/73).

### Genotyping of *CD209* rs4804803 SNP

Genomic DNA was isolated from heparin-anticoagulated blood samples using a standard phenol-chloroform extraction followed by 70% alcohol precipitation. Genotyping for the *CD209* variant (−336 A/G; rs4804803) was carried out using Custom TaqMan SNP Genotyping Assays (Applied Biosystems, Foster City, CA, USA). The primer sequences were 5′-GGACAGTGCTTCCAGGAACT-3′ (forward) and 5′-TGTGTTACACCCCCTCCACTAG-3′ (reverse). The TaqMan minor groove binder probe sequences were 5′-TACCTGCCTACCCTT G-3′, and 5′-CTGCCCACC CTTG-3′. The probes were labeled with the TaqMan fluorescent dyes VIC and FAM, respectively. The PCR was conducted in total volume of 15 µL using the following amplification protocol: denaturation at 95°C for 10 min, followed by 40 cycles of denaturation at 94°C for 20 s, followed by annealing and extension at 60°C for one minute. After the PCR, the genotype of each sample was determined by measuring the allele-specific fluorescence in the ABI Prism 7500 Sequence Detection System, using SDS 1.1 software for allele discrimination (both Applied Biosystems). To validate the genotyping by real-time PCR analysis, 100 PCR products were subject to restriction fragment length polymorphism (RFLP) analysis with MscI restriction enzyme (New England Biolabs, Beverly, MA, USA) and showed 100% identical result between these two genotyping systems.

### Generation of DCs from individuals with AA or AG phenotype of rs4804803

Peripheral blood mononuclear cells were collected from peripheral blood of 20 healthy, DEN-specific IgM or IgG seronegative volunteers with AA or AG genotype. CD14^+^ monocytes were isolated by positive selection according to the manufacturer's specifications using CD14 microbeads and a magnetic cell separator (MACS) (Miltenyi Biotec, Bergisch Gladbach, Germany). Enriched CD14^+^ cells (purity>95%) were cultured for 6 days in six-well plates in complete RPMI medium in the presence of 10 ng/mL rhGM-CSF and 5 ng/mL rhIL-4 at 37°C, and 5% CO_2._ On day 3, half of the medium was replaced with fresh medium supplemented with rhGM-CSF and rhIL-4. Expression of markers was measured by flow cytometer using specific antibodies and their corresponding isotype controls.

### DCs infection with DEN-2 and endogenous IP-10 neutralizing experiments

Unless otherwise stated, monocyte-derived dendritic cells (MDDCs) were infected with DEN-2 at a multiplicity of infection (MOI) of 5 for 2 h at 37°C and 5% CO_2_. Cells were washed twice to remove cell-free virus, and cultured in complete RPMI medium (without cytokines) at a density of 2×10^5^ cells/ml in 48-well plates. Cells and supernatants were removed and analyzed at 24, 48, and 72 h post-infection. For the neutralization experiments, cells were incubated in the medium alone or in the medium with the addition of anti-human CXCL10/IP-10 antibody (R&D Systems, Minneapolis, MN, USA) at 10 µg/mL for 30 min. Cells and supernatants were harvested and analyzed 24 h post-infection.

### Detection of *CD209* mRNA by real time RT-PCR and cell surface expression on MDDCs by flow cytometry

Total RNA extracted from MDDCs was subjected to quantitative RT-PCR to assess levels of mRNA corresponding to *CD209* and *ß2–microglobulin* (*B2MG*) using the ABI PRISM 7500 instrument (Applied Biosystems). The forward primer, reverse primer sequence for detecting *CD209* and *B2MG* were 5′-AACAGCTGAGAGGCCTTGGA-3′, 5′-GGGACCATGGCCAAGACA-3′, and 5′-AATTGAAAAAGTGGAGCATTCAGA-3′, 5′-GGCTGTGACAAAGTCACATGGTT-3′, respectively. The PCR cycling parameters were 40 cycles of PCR reactions at 94°C for 20 s, and 60°C for one minute. The results were detected in real-time and recorded on a plot showing fluorescence *vs.* time. RT-PCR products were also visualized on ethidium bromide-stained 1.5% agarose (Pierce Co., Rockford, IL, USA) gel with a 100- bp ladder (Pharmacia Biotech, Piscataway, NJ, USA) as a reference.

To measure the CD209 cell surface expression, MDDCs were stained with FITC-conjugated mAbs specific for DC-SIGN (R&D Systems, Minneapolis, MN, USA). An isotype-matched FITC-labeled control, mouse IgG2b (clone MOPC195, Immunotech, Beckman Coulter, Fullerton, CA, USA) was included in each experiment.

### Quantitation of viral replication in MDDCs by real time RT-PCR

Total RNA extracted from MDDCs was subjected to assess DEN-2 RNA viral copies. Fluorescent RT-PCR was performed in an ABI 7500 quantitative PCR machine (Applied Biosystems) for 40 cycles using TaqMan technology as previously described [21].

### Detection of cytokine/chemokine production by ELISA

Cytokine/chemokine production and viral replication were determined at 24, 48, and 72 h post-infection. Cell-free culture supernatants TNFα and macrophage chemoattractant protein 1 (MCP-1) concentrations were measured using ELISA kits from eBioscience Inc. (San Diego, CA, USA); IL-12p40 and IFN-inducible protein 10 (IP-10) concentrations were measured using ELISA kits from R&D Systems as per manufacturer's instructions.

### Statistical Analyses

Data are presented as mean ± SEM values. Alleles and genotypes distribution of rs4804803 are presented as numbers (percentages). Conformance of the allele frequencies to Hardy-Weinberg equilibrium proportions was tested to compare the observed and expected frequencies of heterozygotes and homozygotes. Differences among patients with DEN, DF, DHF, OFI, and population controls were determined using two-sided Chi-Square test or Fisher exact test. Odds ratio (OR) values were calculated with 95% confidence intervals (CI). The Student's t-test or Mann-Whitney U test was used for statistical comparisons between continuous variables. The Wilcoxon signed-rank test was used for statistical comparison of the neutralization experiments. All analyses were performed using SPSS 13.0 (SPSS Inc. Chicago, IL, USA).

## Results

### Demographic characteristics of patients with DF and DHF

During a large DEN-2 outbreak in southern Taiwan between June 2002 and January 2003, a hospital-based case-control study was used to identify the risk immune parameters [19]–[23]. Employing the decoded DNA samples from that same cohort of the population that study has been extended to investigate the association of rs4804803 SNP with DF, DHF, viral replication, and immune response. Based on the previous case-control study design, we have included DNA samples from 135 DHF, 176 DF, and 143 OFI patients in this expanded study. The main characteristics of the study population are summarized in Table 1. There were no significant differences in sex or total leukocyte counts between patients with DF and those with DHF. However, age (41.7±1.6 years *vs.* 45.7±1.3 years, *p*<0.001), serum GOT levels (70.1±8.1 U/mL *vs.* 313.8±74.6 U/mL, *p* = 0.002) and GPT levels (67.1±11.3 U/mL *vs.* 142.7±21.9 U/mL, *p* = 0.003) were significantly higher in the DHF group (Table 1). A patient manifested with abdominal pain had ascites as evidenced by abdominal ultrasonography was classified as DF because the patient revealed no thrombocytopenia (<100,000/mm^3^), petechia or hemorrhagic manifestation during the admission period.

**Table 1:** Demographic data of dengue patients with DF and DHF.

	DF (n = 176)	DHF (n = 135)	*p* value
Age (years)	41.7±1.6	45.7±1.3	<0.001
Sex (male/female)	86/90	60/75	0.439
Hematocrit (%)	37.0±0.1	39.7±3.0	0.021
Hemoconcentration# (%)	6.0±0.6	20.7±1.3	<0.001
Platelet (×10^4^/mm^3^)	10.9±0.7	2.6±0.3	<0.001
WBC (/mm^3^)	4,130±190	4,374±238	0.425
GOT (U/mL)	70.1±8.1	313.8±74.6	0.002
GPT (U/mL)	67.1±11.3	142.7±21.9	0.003
Serum albumin level (g/dL)	3.7±0.2	3.1±0.1	0.001
Pleural effusion (%)	0/59 (0)	57/75 (76)	<0.001
Ascites (%)	1/68 (1)	47/85 (55)	<0.001
Secondary infection (%)	63/173 (36)	78/120 (65)	<0.001

*Values are the mean ± SEM or number (%). *P* values were determined by application of Student's *t*-test for continuous variables and by *X^2^* test or Fisher exact test for categorical variables.

# Defined as a 20% increase in hematocrit between the highest and lowest levels.

### *CD209* −336 A/G polymorphisms (rs4804803) associated with dengue infection and DHF susceptibility

We investigated the association of rs4804803 SNP in the promoter region of *CD209* with protection from dengue infection and the susceptibility of DHF. Genomic DNA obtained from DEN patients (n = 311), OFI patients (n = 143), and population controls (n = 120) was genotyped for rs4804803 SNP. We found that GG/AG genotypes in 16.0% of the DEN patients were significantly higher than OFI patients (5.6%, OR = 3.23, *p* = 0.002) and population controls (7.5%, OR = 2.36, *p* = 0.020; Table 2). Moreover, the GG/AG genotypes were significantly higher in DHF patients (23.0%) than OFI patients and population controls (OR = 5.03 and 3.68, *p* = 3×10^−5^ and 0.001), and also significantly higher than DF patients (10.8%, OR = 2.46; *p* = 0.004; Table 2).

**Table 2:** Distribution of *CD209* −336 A/G (rs4804803) genotype in patients with dengue infection (DEN) and other non-dengue febrile illnesses (OFI) or population controls.

	Genotype distribution			Case vs. OFI		Cases vs. Control		DHF vs. DF	
	GG	AG	AA	*OR* ( 95% CI)	*p*	*OR* ( 95% CI)	*p*	*OR* ( 95% CI)	*p*
OFI (143)	0 (0)	8 (5.6)	135 (94.4)	1					
Control (120)	0 (0)	9 (7.5)	111 (92.5)	1.37 (0.51–3.66)	0.531	1			
DEN (311)	2 (0.6)	48 (15.4)	261 (83.9)	3.23 (1.49–7.02)	0.002	2.36 (1.12–4.97)	0.020		
DF (176)	0 (0)	19 (10.8)	157 (89.2)	2.04 (0.87–4.81)	0.097	1.49 (0.65–3.42)	0.342	1	
DHF (135)	2 (1.5)	29 (21.5)	104 (77.0)	5.03 (2.22–11.40)	3×10^−5^	3.68 (1.67–8.09)	0.001	2.46 (1.32–4.59)	0.004

Values are number (%) studied. *P* value was determined by *X^2^* test or Fisher exact test based on GG or GA vs. AA comparison OR, odds ratio. CI, confidence interval.

Analysis of the allele distribution between DEN and OFI patients or population controls showed that the G allele frequency was higher in DEN patients (8.4%), compared with OFI patients (2.8%, OR = 3.17, *p* = 0.002) or population controls (3.8%, OR = 2.34, *p* = 0.018; Table 3). Moreover, the frequency of G allele of rs4804803 was significantly higher in DHF patients (12.2%) than OFI patients or population controls (OR = 4.84 and 3.57, *p* = 2×10^−5^ and 0.001), and higher than DF patients (5.4%, OR = 2.44, *p* = 0.002; Table 3). Few DHF patients (n = 6) had dengue shock syndrome in this cohort; one of them carrying AG genotype.

**Table 3:** Distribution of *CD209* −336 A/G (rs4804803) allele in patients with dengue infection (DEN) and other non-dengue febrile illnesses (OFI) or population controls.

	Allele distribution		Cases vs. OFI		Cases vs. Control		DHF vs. DF	
	G	A	*OR* ( 95% CI)	*p*	*OR* ( 95% CI)	*p*	*OR* ( 95% CI)	*p*
OFI	8 (2.8)	278 (97.2)	1					
Control	9 (3.8)	231 (96.2)	1.35 (0.51–3.57)	0.538	1			
DEN	52 (8.4)	570 (91.6)	3.17 (1.49–6.77)	0.002	2.34 (1.14–4.83)	0.018		
DF	19 (5.4)	333 (94.6)	1.98 (0.86–4.60)	0.105	1.46 (0.65–3.29)	0.354	1	
DHF	33 (12.2)	237 (87.8)	4.84 (2.19–10.68)	2×10^−5^	3.57 (1.67–7.63)	0.001	2.44 (1.36–4.40)	0.002

Values are number (%) studied. *P* value was determined by *X^2^* test or Fisher exact test OR, odds ratio. CI, confidence interval.

### No association of *CD209* −336 A/G polymorphism (rs4804803) with primary and secondary dengue infection

To investigate whether the rs4804803 SNP in the promoter region of *CD209* associated with the primary and secondary DEN infection, we used serological methods to detect DEN antibodies for differentiation into primary and secondary dengue infection. Of the 293 DEN patients, 141 (48%) had secondary DEN infections, based on detectable DEN-2 virus RNA and DEN IgG. As shown in Table 1, secondary DEN-2 infection was more frequently found in patients with DHF than in those with DF (65% vs. 36%, *p*<0.001). We found the rs4804803 GG/AG genotypes were found in 12.5% of patients with primary DEN infection and 16.3% of patients with secondary DEN infection, which did not reach significantly different (OR = 1.36; *p* = 0.352). As shown in Table 4, there was no association between rs4804803 SNP and primary or secondary dengue infection in DF patients (OR = 0.64; *p* = 0.409), or in DHF patients (OR = 1.80; *p* = 0.251). In addition, there was no association between allele distribution and primary or secondary dengue infection in DF and DHF patients (data not shown).

**Table 4:** Distribution of *CD209* −336 A/G genotype in patients with primary and secondary dengue infection.

	DF					DHF				
	GG	AG	AA	*OR* ( 95% CI)	*p*	GG	AG	AA	*OR* ( 95% CI)	*p*
Primary (152)	0 (0)	13 (11.8)	97 (88.2)	1		0 (0)	6 (14.3)	36 (85.7)	1	
Secondary (141)	0 (0)	5 (7.9)	58 (92.1)	0.64 (0.22–1.90)	0.409	2 (2.6)	16 (20.5)	60 (76.9)	1.80 (0.65–4.95)	0.251

Values are number (%) studied. *P* value was determined by *X^2^* test or Fisher exact test based on GG or GA vs. AA comparison. OR, odds ratio. CI, confidence interval. Primary and secondary dengue infections are defined by detectable DEN-specific IgM and IgG, respectively, within one week of the illness.

### Increase of DC-SIGN expression on MDDCs from individuals with AG genotype of rs4804803

**Figure 1:**
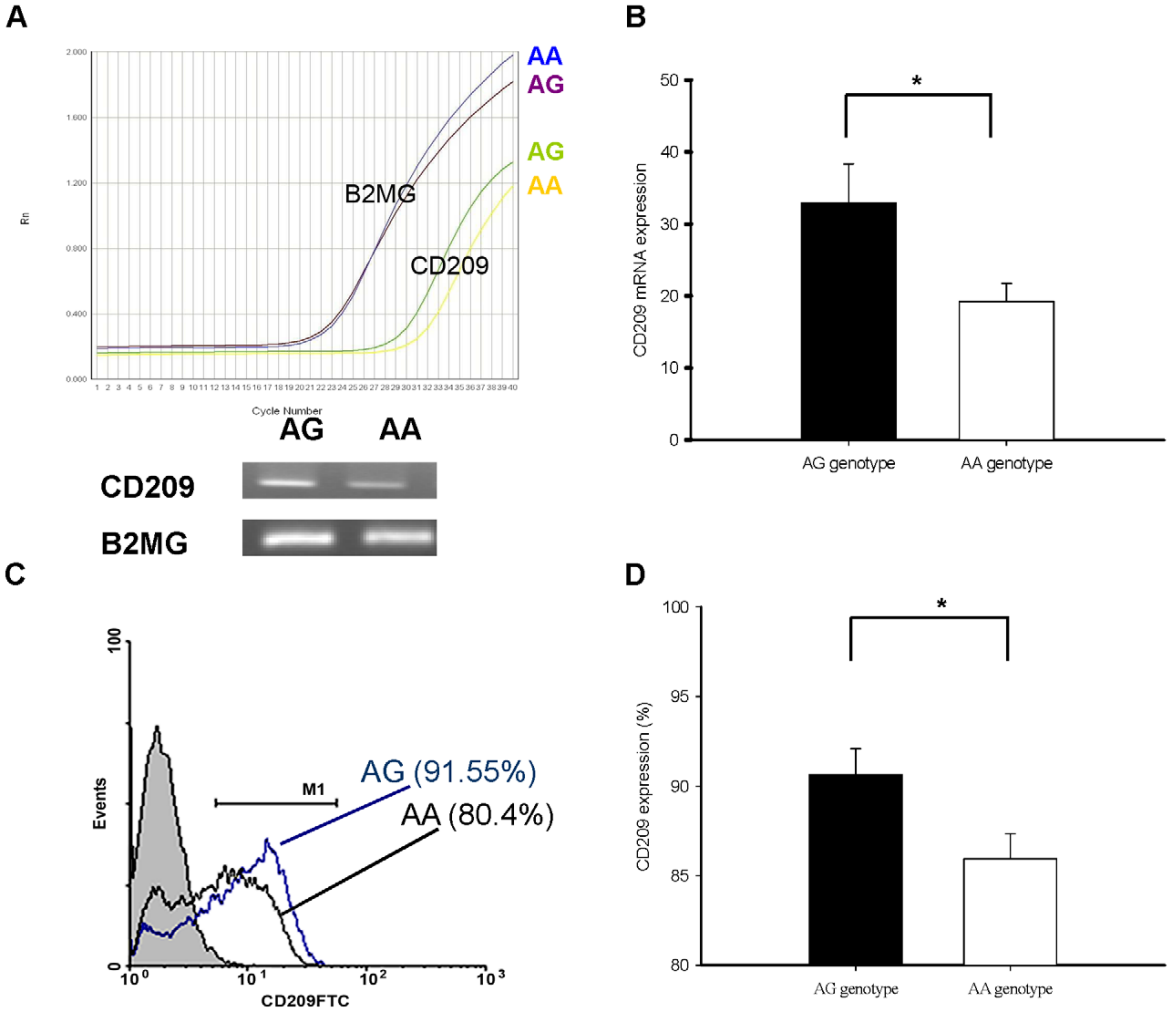
*CD209* mRNA and cell surface expression on MDDCs with AA or AG genotype of rs4804803. (A) Quantitative RT-PCR analysis of *CD209* mRNA in MDDCs was run parallel to *ß2-microglobulin* (*B2MG*) mRNA expression (control) on the real-time RT-PCR tracing and the gel view of RT-PCR products. (B) Subjects with rs4804803 AG genotype had higher *CD209* mRNA expression in MDDCs than those with AA genotype (*p* = 0.032). (C) Flow cytometric analysis of CD209 surface expression on MDDCs from individuals with the rs4804803 AG genotype (blue line; MFI = 34.1) and with the AA genotype (black line; MFI = 30.4). (D) MDDCs from individuals with AG genotype had significantly higher CD209 cell surface expression than those with AA genotype (*p* = 0.029). The results are shown as mean ± s.e.m. from subjects with AA (n = 10) or AG (n = 10) genotype in three independent experiments. Statistical values were determined by Mann-Whitney U test. Asterisk (*) indicates a significant difference (*p*<0.05).

**Figure 2:**
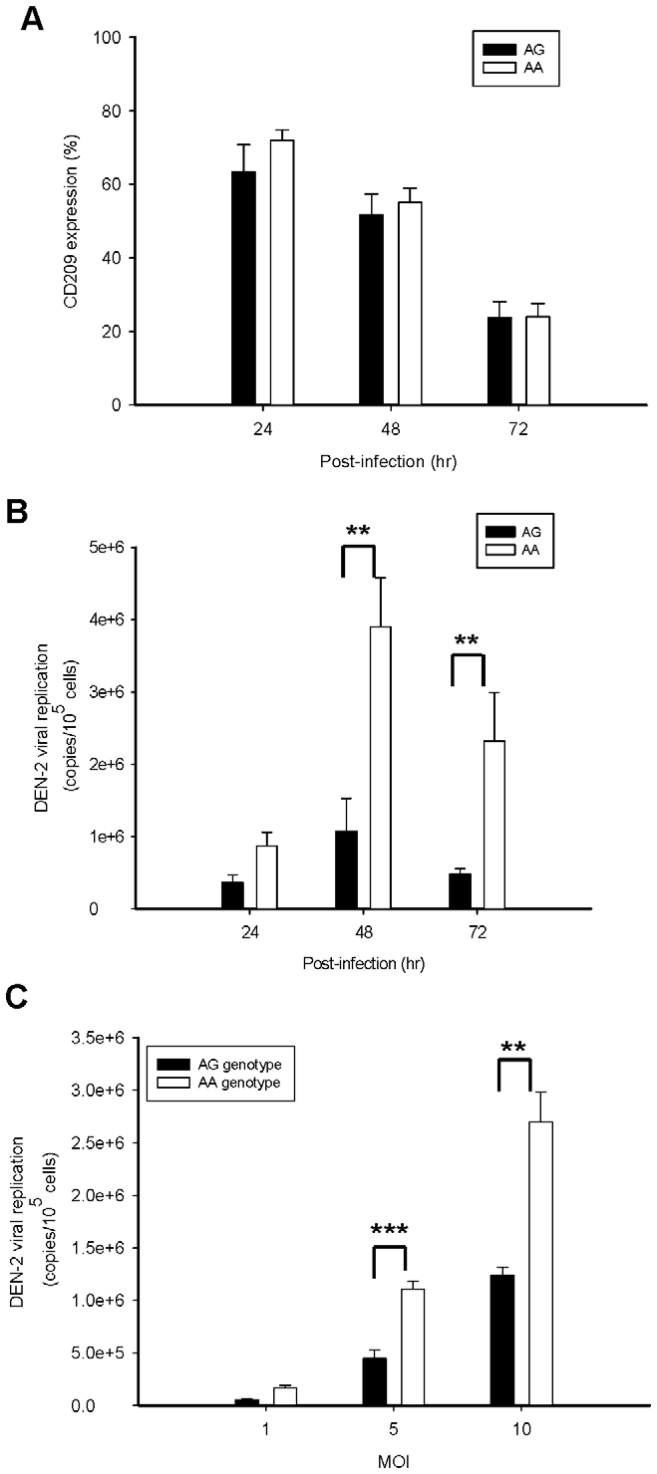
Kinetic surface CD209 expression and viral replication from DEN-infected MDDCs with AA or AG genotype. (A) Kinetic CD209 surface expression on MDDCs from individuals with AA or AG genotype of rs4804803 by flow cytometry. The surface CD209 expression declined along with infection in subjects with both genotypes. (B) Kinetic changes of DEN-2 replication (virus copies per 10^5^ cells) were assessed after infection at MOI of 5 for 24 to 72 h. Viral replication was significantly higher in MDDCs from individuals with rs4804803 AA genotype than those with AG genotype at 48 hr and 72 hr post-infection (*p* = 0.006 and 0.003, respectively) The results are shown as mean ± s.e.m. from subjects with AA (n = 8) or AG (n = 8) genotype in three independent experiments. (C) Changes in the yield of DEN-2 replication (virus copies per 10^5^ cells) were assessed at MOIs of 1, 5, and 10 at 72 h post-infection. Viral replication was significantly higher in MDDCs from individuals with AA genotype than those with AG genotype at MOIs of 5 and 10 (*p*<0.001 and 0.002, respectively). The results are shown as mean ± s.e.m. from subjects with AA (n = 10) or AG (n = 10) genotype in three independent experiments. Statistical values were determined by Mann-Whitney U test. Asterisk (*) indicates a significant difference (*p*<0.05). ** indicates *p*<0.01; *** *p*<0.001.

Due to the low frequency of GG genotype in our population (2 cases, 0.6%), we could not recall the patients because the data file had been decoded for identification. We examined DC-SIGN (CD209) expression in both mRNA level of MDDCs and protein level on their cell surface from healthy subjects with AA or AG genotype by quantitative RT-PCR and flow cytometry, respectively. A significant increase in *CD209* mRNA expression was detected in the MDDCs from individuals with AG genotype than those from individuals with AA genotype (*p* = 0.032, Fig 1B). Similarly, individuals with AG genotype had a significantly higher cell surface DC-SIGN expression (*p* = 0.029; Fig 1D). However, the surface DC-SIGN expression declined rapidly along with DEN-2 infection on MDDCs from both genotypes' subjects, which showed no difference at 24, 48, and 72 h post-infection (Fig. 2A).

### Lower viral replication in MDDCs from individuals with AG genotype of rs4804803

To investigate whether the rs4804803 SNP was correlated to viral replication, we measured DEN-2 RNA copies in MDDCs with AA or AG genotype of rs4804803 at 24, 48, and 72 h post-infection. DEN-2 replication was significantly higher in MDDCs from individuals with AA genotype than those with AG genotype at 48 h post-infection (1.07±0.45×10^6^ copies/10^5^ cells *vs.* 3.90±0.67×10^6^ copies/10^5^ cells, *p* = 0.006) and 72 h post-infection (4.83±0.70×10^5^ copies/10^5^ cells *vs.* 2.32±0.68×10^6^ copies/10^5^ cells, *p* = 0.003; Fig. 2B). Viral replication, as measured at 72 h post-infection, increased more remarkably in MDDCs at MOI of 5 and 10 (*p*<0.001 and 0.002, respectively; Fig. 2C).

### Elevation of cytokines TNFα, IL-12p40, and chemokine IP-10 production in DEN-infected MDDCs with AG genotype of rs4804803

**Figure 3:**
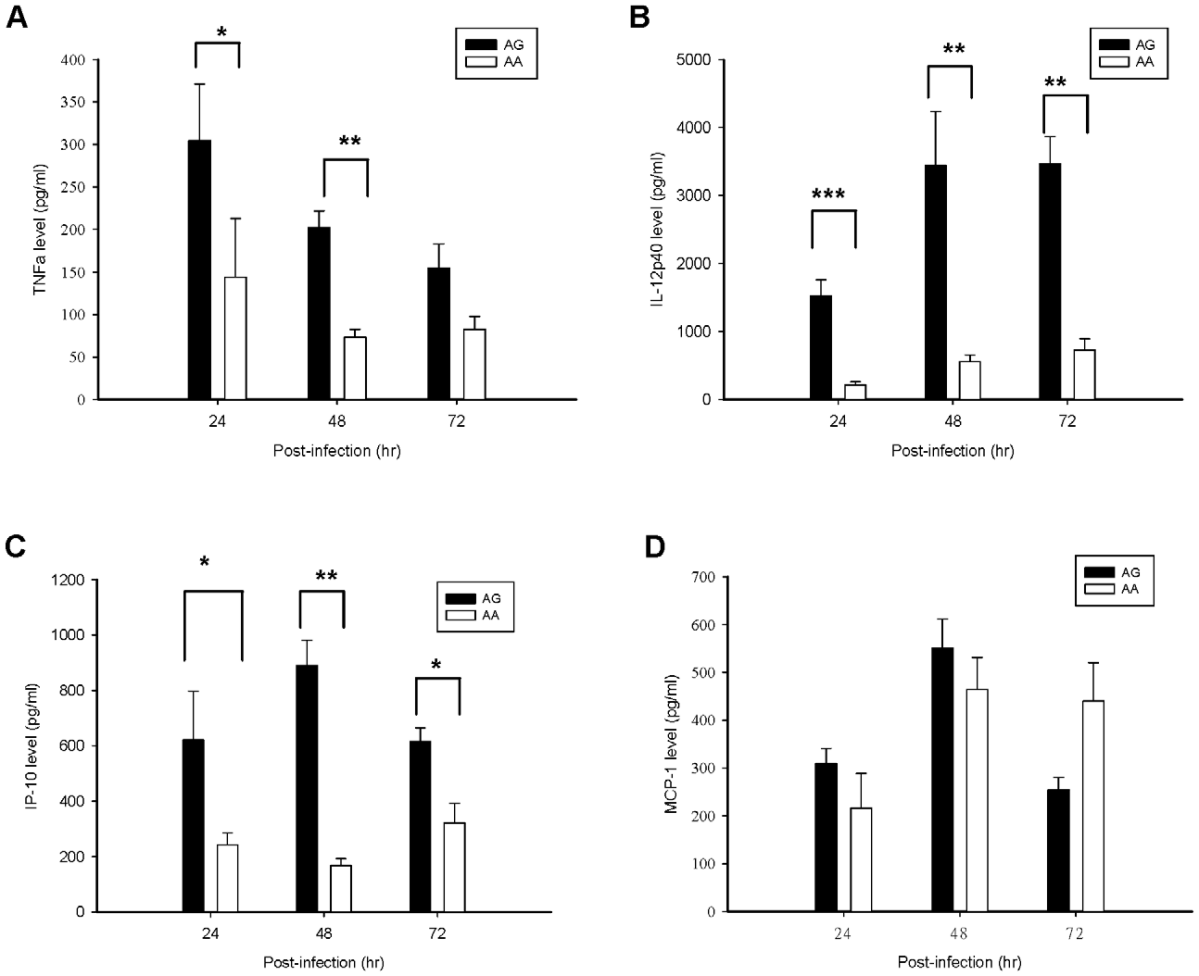
Kinetic cytokine/chemokine production by DEN-infected MDDCs from individuals with AA or AG genotype of rs4804803. The supernatant of TNFα, IL-12p40, IP-10 and MCP-1 levels were measured using ELISA after DEN infection at MOI of 5 for 24 to 72 h. The production of TNFα (A), IL-12p40 (B), and IP-10 (C) levels after DEN-2 infection was significantly higher in MDDCs from individuals with AG genotype than those with AA genotype. The production of MCP-1 (D) after DEN-2 infection was not significantly different between the two groups. The results are shown as mean ± s.e.m. from subjects with AA (n = 8) or AG (n = 8) genotype in three independent experiments. Statistical values were determined by Mann-Whitney U test. Asterisk (*) indicates a significant difference (*p*<0.05). ** indicates *p*<0.01; *** *p*<0.001.

To investigate whether higher cell surface DC-SIGN expression was correlated with immune response, we investigated kinetic cytokine/chemokine production by MDDCs from individuals with AA or AG genotype of rs4804803. Results showed that MDDCs with AG genotype had significantly higher TNFα production than those with AA genotype at 24 and 48 hr post-infection (303.51±66.75 pg/mL *vs.* 143.97±68.80 pg/mL and 202.35±19.35 pg/mL *vs.* 73.00±9.55 pg/mL; *p* = 0.021 and 0.002, respectively; Fig 3A). IL-12p40 production significantly increased by MDDCs with AG genotype than those with AA genotype at 24, 48, and 72 h post-infection (*p*<0.001, 0.007 and 0.001, respectively; Fig 3B). We also measured the concentration of two chemokines, MCP-1 and IP-10, which had been implicated in the recruitment and stimulation of monocytes, macrophages, dendritic cells, NK cells, and T lymphocytes [25]. It was found that IP-10, but not MCP-1, production was significantly higher by MDDCs with AG genotype than those with AA genotype at 24, 48, and 72 h post-infection (620.60±175.56 pg/mL *vs.* 243.02±41.64 pg/mL, 889.92±91.46 pg/ml *vs.* 168.02±24.02 pg/mL, and 614.44±49.16 pg/mL *vs.* 322.32±69.62 pg/mL; *p* = 0.034, 0.009 and 0.010, respectively; Fig 3C). The MCP-1 levels peaked at 48 hr in subjects with both genotypes', but there was no significant difference between AA genotype and AG genotype (550.72±60.73 pg/mL *vs.* 463.92±66.80 pg/mL, *p* = 0.157; Fig. 3D).

### IP-10 production by MDDCs involved in viral replication of DEN infection

**Figure 4:**
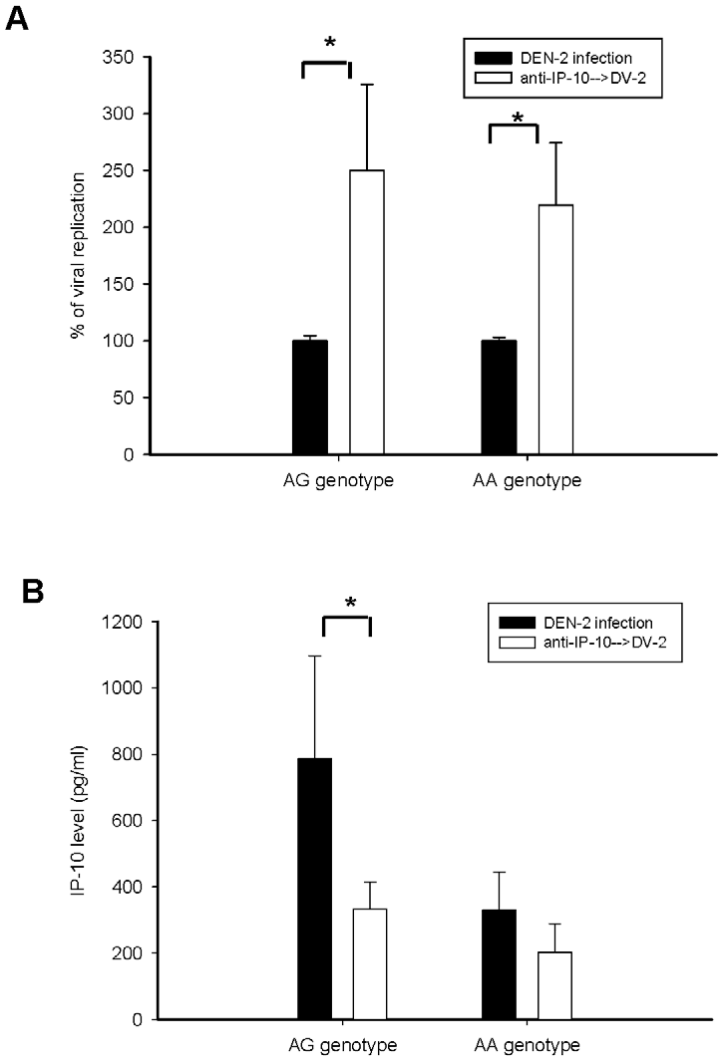
IP-10 production by MDDCs involved in the viral replication of DEN infection. MDDCs were mock-infected or DEN infected (MOI = 5) after preincubation, with and without anti-IP-10 neutralizing antibody (10 µg/mL) for 30 min. Total RNA was extracted from MDDCs and the supernatants were collected 24 h post-infection. The percentage of viral replication (A) and the production of IP-10 levels (B) following DEN-2 infection were significantly higher by MDDCs from individuals with AG genotype than from individuals with AA genotype. Following DEN-2 infection, the production of IP-10 by MDDCs from individuals with AA genotype was not significantly different between those with and without anti-IP-10 neutralizing antibody. The results are shown as mean ± SEM from three independent experiments (n = 7). Statistical values were determined by Wilcoxon signed-rank test. Asterisk (*) indicates a significant difference (*p*<0.05).

IP-10, produced by non-infected bystander DCs in response to DEN infection, is a potent chemoattractant for activated T and NK cells [26], and the modulation of adaptive immune response [27]. IP-10 has also been known to inhibit the binding ability of DEN in immortalized cells [28]. In our MDDC model, cells from individuals with AG genotype exhibited an augmented innate immune reaction, showing higher IP-10 production, post-infection (Figure 3C). Based on these results, we hypothesized that DEN-infected MDDCs with AG genotype produced higher levels of IP-10, which might block viral entry or viral replication in MDDCs. We used an anti-IP-10 neutralizing mAb to block endogenous IP-10 production by MDDCs. With both genotypes, the viral replication 24 h post DEN infection increased significantly more in the presence of neutralizing antibody than in the absence of neutralizing antibody (*p* = 0.034 and 0.040, respectively; Fig. 4A). IP-10 production by MDDCs from individuals with AG genotype significantly decreased (795.3±368.1 pg/mL *vs.* 273.8±87.8 pg/mL; *p* = 0.037), but it did not decrease in MDDCs from individuals with AA genotype (329.8±114.2 pg/mL *vs.* 201.8±87.0 pg/mL; *p* = 0.091; Fig. 4B). These results suggest that IP-10 produced by MDDCs is involved in the viral replication of DEN infection.

## Discussion

DC-SIGN has been shown to be an important receptor for DEN and a number of viruses, including HIV, *Helicobacter pylori*, and *Mycobacterium tuberculosis* and hepatitis C virus (HCV) [29]. Some studies have demonstrated that genetic variations of *CD209* (rs4804803) were associated with the susceptibility to HIV [14], *Mycobacterium tuberculosis* [17], HCV [30], and dengue [16]. Few studies have demonstrated how the rs4804803 SNP is involved in viral replication or immune response. We are the first in the field to demonstrate the relationship among functional cell surface expression, viral replication, and immune responses in DEN-infected MDDCs from subjects with rs4804803 SNP. Here we found that rs4804803 SNP was strongly associated with the risk of DHF vs. DF and controls. Functional studies have determined that MDDCs from individuals with AG genotype have a significantly higher cell surface DC-SIGN expression than from those with AA genotype. MDDCs with AG genotype produced higher TNFα, IL-12p40, and IP-10 levels but lower viral replication in response to dengue infection.

Because the physiopathology of various manifestations of DHF is not fully understood, several studies have supported the supposition that secondary dengue infection [31], age [32], a number of preexisting chronic diseases such as diabetes and bronchial asthma [33], and host genetic factor [16], [22] increase the risk of progression to DHF. This indicates that multiple factors are involved in the development of DHF. Our findings regarding rs4804806 SNP associated with DHF vs. OFI control (*p* = 3×10^−5^; Table 2) in a case-control association study suggests that rs4804806 SNP contributes in part to the development of DHF.

Our study shows that the GG/AG genotypes of rs4804803 were associated with susceptibility to DHF, compared with DF, which is consistent with the observation of Sakuntabhai *et al.* [16]. In our study, the AA genotype was associated with protection against DHF, compared with OFI and population controls, while G allele was associated with protection against DF in Sakuntabhai's observation. The inconsistency between these studies regarding the protection for DHF or DF may result from two possibilities. First, the frequency of G allele in Chinese population is 3.8%; while in Thailand, it is 9.5–10.4% [16], [34]. Second, definition of DF and DHF might be also different. We defined DF and DHF according to WHO criteria, while in the study by Sakuntabhai *et al.*, DF was defined by criteria of severe dengue fever syndrome with hemorrhage but no plasma leakage, excluding patients with flu-like symptoms or those having only fever. Moreover, the rs4804803 SNP was demonstrated to be in linkage disequilibrium with three other intronic polymorphisms in a Thai population, and these might also have contributed to the susceptibility of DHF [16].

Our results suggest that humans carrying the rs4804803 AG genotype have a higher DC-SIGN expression and lower DEN-2 replication in MDDCs. These results differ from a previous study by Loach *et al.* who demonstrated that the DC-SIGN expression levels on Raji cells after transfection of various DC-SIGN cDNA constructs were significantly correlated to the infection rate of DEN-1 [35]. DC-SIGN is an endocytic receptor shown to induce endocytosis of several pathogens, including dengue [36]–[38]. The difference between these two studies might be due to different cell types and *ex vivo* culture systems. In our study, it was found that MDDCs from subjects with rs4804803 AG genotype had higher surface DC-SIGN expression with higher production of chemokines such as IP-10, which could limit DEN-2 replication (Fig. 4A); however, the higher surface DC-SIGN expression in subjects with AG genotype decreased remarkably 24 h post-infection (Fig. 2A). In the study by Loach *et al.*, ectopic expression levels of DC-SIGN on Raji cells enhanced DEN-1 replication, which might be related to a higher quantity of receptors or lower production of IP-10 favoring DEN replication. Results from these studies suggest that the correlation of viral replication to higher or lower DC-SIGN expression depends on genetic factors in the host, cell type, and dynamic changes in the receptor following DEN infection.

In functional studies of rs4804803 SNP, we determined that MDDCs with AG genotype had a higher DC-SIGN expression correlated to augmented immune responses with higher TNFα, IL-12p40, and IP-10, than those with AA genotype, but not MCP-1 production. DEN replication was significantly lower in individuals with AG genotype. The addition of anti-IP-10 neutralizing antibody blocked the production of endogenous IP-10 and significantly enhanced the replication of DEN-2 (Fig. 4A). This suggests that rs4804803 SNP was involved in the DC-SIGN expression associated with augmented immune response, such as the increase in the production of IP-10 that repressed the replication of DEN. This is supported by the fact that altered immune response, but not viral load, was observed in DHF patients [21], [39]. CLEC5A-mediated DEN infection in animals that was susceptible to DEN hemorrhagic infection also revealed augmented immune response [40].

In contrast, it is interesting to note that the viral replication in MDDCs from individuals with rs4804803 AA genotype was significantly higher than in individuals with AG genotype following DEN-2 infection. The mechanism by which rs4804803 SNP influences DEN replication in MDDCs is currently unknown. Chan *et al.* showed that certain polymorphisms of *L-SIGN*, a *DC-SIGN* homologue, mediated more efficient viral degradation of SARS-CoV [41]. The clinical implications of screening genotypes to prevent DEN infection might be supported if different viral loads could be demonstrated among humans with various genotypes of rs4804803 in future outbreaks of DEN.

The outcome of DEN infection is determined by a myriad of interactions among viral, immunological, and human genetic factors, as well as kinetic interactions between innate and adaptive immunity. This study provides new evidence that *CD209* rs4804803 SNP, correlated to cell surface expression on dendritic cells, mediates augmented immune responses against DEN-2 infection and is implicated in the susceptibility of DHF. Further studies are warranted, particularly with regard to the genetic variants of *CD209* on the DC polarization of adaptive immunity, and how they may promote or protect the development of DHF.
